# The MSHA Strain of *Pseudomonas Aeruginosa* Activated TLR Pathway and Enhanced HIV-1 DNA Vaccine Immunoreactivity

**DOI:** 10.1371/journal.pone.0047724

**Published:** 2012-10-15

**Authors:** Jue Hou, Yong Liu, Ying Liu, Yiming Shao

**Affiliations:** State Key Laboratory for Infectious Disease Prevention and Control, National Center for AIDS/STD Control and Prevention, Chinese Center for Disease Control and Prevention, Beijing, China; Harvard Medical School, United States of America

## Abstract

The mannose-sensitive hemagglutination pilus strain of *Pseudomonas aeruginosa* (PA-MSHA) has been shown to trigger naïve immune responses through the activation of monocytes, macrophages, natural killer cells (NK cells) and antigen presenting cells (APCs). Based on the hypothesis that PA-MSHA activates natural immunity through the Toll-like receptor (TLR) pathway, we scanned several critical TLR pathway molecules in mouse splenocytes using high-throughput real-time QRT-PCR and co-stimulatory molecule in bone marrow-derived dendritic cells (BMDCs) following *in vitro* stimulation by PA-MSHA. PA-MSHA enabled activation of the TLR pathway mediated by NF-κB and JNK signaling in splenocytes, and the co-stimulatory molecule CD86 was up-regulated in BMDCs. We then assessed the adjuvant effect of PA-MSHA for HIV-1 DNA vaccines. In comparison to DNA inoculation alone, co-inoculation with low dosage of PA-MSHA enhanced specific immunoreactivity against HIV-1 Env in both cellular and humoral responses, and promoted antibody avidity maturation. However, high doses of adjuvant resulted in an immunosuppressive effect; a two- or three-inoculation regimen yielded low antibody responses and the two-inoculation regimen exhibited only a slight cellular immunity response. To our knowledge, this is the first report demonstrating the utility of PA-MSHA as an adjuvant to a DNA vaccine. Further research is needed to investigate the exact mechanisms through which PA-MSHA achieves its adjuvant effects on innate immune responses, especially on dendritic cells.

## Introduction

Despite decades of global research efforts, an efficacious HIV vaccine has remained elusive thus far. Plasmid DNA vaccines are a promising modality for immunization against a variety of human pathogens. However, poor delivery efficiency has impaired their practical use; despite considerable efforts to improve delivery, DNA vaccination results in only minute levels of antigens in the body for inducing the immune system. Consequently, a number of adjuvant strategies have been designed to improve plasmid DNA immunogenicity, including directly stimulating the immune system as well as enhancing plasmid DNA expression.

DNA vaccine adjuvants are an active field of research and have generated a broad range of candidate molecules. CpG oligodeoxynucleotide (CpG-ODN), a successful adjuvant, has been shown in several clinical trials [1], [2], [3] and pilot studies [4], [5], [6], [7] to effectively enhance specific cellular and humoral immune responses. In addition, other materials such as bacterial toxins [8], saponins [9], lipopolysaccharide derivatives [10], lipopeptides and cytokines have also demonstrated adjuvant effects.

In addition, an increasing number of studies have demonstrated the adjuvant effects of flagellin [11], [12], [13], [14], [15], [16], including its ability to promote cytokine production through generalized recruitment of T and B lymphocytes and to activate dendritic cells and T lymphocytes through the Toll-like receptor (TLR) signaling pathway by the receptor TLR5 [14], [17]. In the mouse model, studies have also found that stimulation with flagellin resulted in substantial activation of murine bone marrow-derived dendritic cells (BMDCs) [18], [19], [20], [21]. Although interactions between flagellin and TLR5 in dendritic cells have been extensively examined [22] and evidence that flagellin stimulates APC activation has been well characterized [23], little is known about the interactions of APCs with other bacterial material involving flagella.

In this study, we examined a variant strain of *Pseudomonas aeruginosa*, PA-MSHA, which has been biologically engineered with low toxicity and lined with fragile and straight MSHA (mannose sensitive hamemagglutination) fimbriae [24], [25]. A previous study demonstrated that heat-killed *P. aeruginosa* can serve as a systemic adjuvant [26] and that the partial adjuvant efficacy of PA-MSHA is due to the fimbriae. PA-MSHA has been shown to activate Th1-type immune responses and has been FDA-approved and used clinically in China in cancer therapies to modulate immune responses. As well, it has been reported to activate innate immunity; stimulate macrophages, natural killer cells and dendritic cells; promote DC maturation and migration; and increase the secretion or expression of cytokines and co-stimulatory molecules such as CD80, CD86, and MHC-II.

Here, we ascertained the ability of PA-MSHA to activate innate immune responses through assessing TLR signaling pathway activation in splenocytes and BMDCs activation following *in vitro* stimulation with PA-MSHA. Furthermore, we evaluated PA-MSHA's ability to augment the cellular or humoral immune responses elicited by various dosages of an HIV-1 *env* DNA vaccine. In our mouse model, the HIV DNA vaccine's immunogenicity was robustly enhanced when co-immunized with PA-MSHA. Interestingly, however, PA-MSHA elicited dose-dependent immune responses at low doses (10^2^–10^4^ CFU) but immunosuppressive responses at high doses (10^8^ CFU). Our data suggests that a suitable dose of PA-MSHA holds promise as an effective adjuvant for enhancing HIV-1 DNA vaccines.

## Materials and Methods

### Adjuvant and DNA vaccine

*Pseudomonas aeruginosa* injection (PA-MSHA) was provided by Beijing Wanteer Bio-Pharmaceutical Co., Ltd. PA-MSHA used in this research was scale-cultured at 37°C for 24 h, inactivated by chemical method and purified by centrifugation, and suspended in phosphate buffered saline (PBS).

Plasmid pGp145_5m_ was constructed by Division of Research on Virology and Immunology, National Center for AIDS/STD Control and Prevention (NCAIDS), China CDC. The plasmid encodes the HIV-1 *env* gene region gp145_5m_ from the HIV-1 strain CN54, and serves as the DNA vaccine.

### Ethics Statement

All experiments were conducted in accordance with the guidelines of Laboratory Animal Center of China CDC and NCAIDS. All procedures involving animal use and care were approved by the NCAIDS Institutional Committee on Laboratory Animals.

### Splenocytes stimulated by PA-MSHA in vitro

6–8 week-old female BALB/c mice were euthanized and their spleens were harvested. RBCs were lysed with RBC lysis buffer according to the protocol described previously [27], [28].

Splenocytes (1×10^6^ cells/well) were then seeded into 96-well plates (Costar, Corning, NY) in RPMI 1640 medium (Invitrogen Corp.) supplemented with 10% fetal bovine serum, 100 IU/ml penicillin, 100 μg/ml streptomycin, 1% L-glutamine and 1 mM HEPES, then incubated in a humidified 5% CO_2_ incubator at 37°C for 3 h. Adjuvant PA-MSHA was then added to the cells at a final concentration of 10^7^ CFU/1×10^6^ cells/ml. Buffer alone was used as the negative control. After 3, 6 and 9 hours, cells were collected by centrifugation and stored at −80°C until assayed. All assays were done in duplicate.

### Profiling of Toll-like receptor-mediated signaling pathway in vitro by real-time RT-PCR

Total RNA from splenocytes, stimulated by PA-MSHA and collected at different time-points, were extracted using RT^2^ qPCR-Grade RNA Isolation Kit (SABiocience, catalog no. PA-001) following the manufacture's protocol. RNA quality and quantity were assessed using a spectrophotometer by measurement of A260/A280. For amplification, 500 µg of total RNA was reverse transcribed to cDNA using RT^2^ First Strand Kit (SABiocience, catalog no. C-03). RT^2^ Profiler™ PCR Array Mouse Toll-Like Receptor Signaling Pathway kit (catalog no. PAMM-018A) was used to evaluate the expression profiles of the Toll-like receptor-mediated naïve immune response.

Quantitative real-time RT-PCR was performed using an ABI Prism 7500 series RT-PCR thermocycler (Applied Biosystems). The threshold cycle (C_T_) was calculated for each gene using the associated Sequence Detection software, version 1.2.2 (Applied Biosystems). The threshold and baseline were set manually according to the manufacturer's instructions. According to a previous study [29], C_T_ data were uploaded into the data analysis template on the manufacturer's website (http://www.sabiosciences.com/pcr/arrayanalysis.php). The relative expression of each gene as compared to control animals was calculated on the website using the ΔΔ C_T_ method with five housekeeping genes as quantification controls (Gusb, Hprt1, Hsp90ab1, Gapdh, Actb).

### Western blot and proteome profiling analysis

Splenocytes were harvested 6 h after stimulation by PA-MSHA and washed with phosphate-buffered saline. Whole cell lysates were obtained by treating cells with RIPA buffer (1% w/w NP-40, 1% w/v sodium deoxycholate, 0.1% w/v SDS, 0.15 M NaCl, 0.01 M sodium phosphate, 2 mM EDTA, and 50 mM sodium fluoride) plus protease inhibitors cocktail set I (Merck Corp.). The total soluble protein concentrations were determined by BCA assay. SDS containing buffer was added to supernatant and heat denature for 10 min before SDS-PAGE loading. After size separation by 12% SDS-PAGE, proteins were transferred onto PVDF membranes by iBot Gel Transfer system (Invitrogen Corp.). Following overnight blocking with 1% (w/v) bovine serum albumin (BSA) in phosphate-buffered saline (PBS) at 4°C, the PVDF membranes were incubated at room temperature for 2 h with rabbit anti-NF-κB, rabbit anti-phospho- NF-κB and anti-β-Actin antibodies, followed by five washing steps and incubation with 1∶3000 PBS-diluted mouse anti-rabbit IgG conjugated to horseradish peroxidase. Reactive bands were visualized using an ECL system (Pierce ECL Western Blotting Substrate).

Mouse-specific cytokines secreted by splenocytes in the presence or absence of PA-MSHA were determined using a mouse cytokine array panel A kit (40 cytokines) (R&D Systems, Abingdon, UK) according to the protocols provided.

### Generation of bone marrow-derived dendritic cells (BMDCs)

Generation of DCs from mouse bone marrow was performed as described previously [30]. Briefly, 6 week old C57BL/6 mice were obtained from Vital River Laboratories (Beijing, China). Bone marrow was flushed from the tibias and femurs, and the RBCs were lysed with hypotonic buffer (9.84 g/L NH_4_Cl, 1 g/L KHCO_3_, and 0.1 mM EDTA). The cells were seeded into 6-well flat-bottom plates (Costar, Corning, NY) in R10 complete medium (RPMI-1640 containing 10% fetus bovine serum, 100 IU/ml penicillin, 100 μg/ml streptomycin, 2 mM glutamine and 1 mM HEPES) containing 20 ng/mL murine granulocyte-macrophage colony stimulating factor (GM-CSF, R&D) and 20 ng/mL recombinant murine interleukin-4 (IL-4, R&D) at 37°C and 5% CO_2_. After 48 h, non-adherent cells were gently removed, and medium containing GM-CSF and IL-4 was replenished. On day 6 of culture, non-adherent and loosely adherent cells were collected as BMDCs.

### Activation of BMDCs by PA-MSHA and FACS analysis

On day 7, FACS analyses revealed 60–70% of BMDCs were mature DCs. BMDCs were then placed in a 6-well culture plate at 1.0×10^6^ cells in 1 ml medium. BMDCs were activated and harvested after 24 h and 48 h of incubation with medium alone (negative control), PA-MSHA (10^3^,10^5^,10^7^ CFU/1.0×10^6^ cells/ml), or *Escherichia coli* lipopolysaccharide (LPS) (Sigma) at final concentration of 10 ng/ml. Activation of BMDCs was measured by FACS analysis.

Expression levels of different surface molecules were assessed by flow cytometry analysis using a FACS Aria cytometer and data was analyzed with FlowJo software. For staining, 2×10^5^ cells were incubated in staining-buffer (PBS and 0.5% BSA) with specific antibodies or the corresponding isotype control (APC-CD11c (clone N418), FITC-CD86 (clone GL1), PE-MHC-I (H-2Kb) (clone AF6-88.5.5.3), all from eBioscience) for 30 min on ice in the dark. Stained cells were centrifuged for 3 min at 2000 rpm and were washed twice with staining-buffer. Baseline fluorescence was measured with unstained cells.

### BMDC endocytosis activity

The BMDC maturation was detected at the endocytosis activity by the take of dextran-FITC (Sigma). BMDC was stimulated for 24 h in the absence or presence of PA-MSHA. Then, BMDCs were suspended in staining buffer (1% fetal bovine serum in PBS) with 200 μg/ml FITC-Dextran and incubated in the dark at 4°C for 1 h to assess non-specific binding or at 37°C to assess specific uptake, after which cells were washed extensively with PBS and analyzed by flow cytometry.

### Immunization of mice

The pGP145_5m_ vaccine was co-formulated with PA-MSHA. Briefly, different concentrations of PA-MSHA (from 10^2^ to 10^8^ CFU/mouse) were premixed with 50 μg DNA vaccine to a final volume of 100 μl each (50 μl for each tibialis anterior muscle) and injected directly. Table 1 shows the immunization timeline and strategies.

**Table 1:** Immunization scheme.

Group	DNA Dosage	PA-MSHA Dosage	Inoculations	Number of mice
1	50 μg	50 μl PBS	2	6
2	50 μl PBS	50 μl(10^2^ CFU)	2	6
3	50 μl PBS	50 μl(10^8^ CFU)	2	6
4	50 μg	50 μl(10^2^ CFU)	2	6
5	50 μg	50 μl(10^4^ CFU)	2	6
6	50 μg	50 μl(10^6^ CFU)	2	6
7	50 μg	50 μl(10^8^ CFU)	2	6
8	50 μg	50 μl PBS	3	6
9	50 μl PBS	50 μl(10^2^ CFU)	3	6
10	50 μl PBS	50 μl(10^8^ CFU)	3	6
11	50 μg	50 μl(10^2^ CFU)	3	6
12	50 μg	50 μl(10^4^ CFU)	3	6
13	50 μg	50 μl(10^6^ CFU)	3	6
14	50 μg	50 μl(10^8^ CFU)	3	6

Six- to eight-week-old female BALB/c mice (Vital River Laboratories) were randomly divided into 14 groups with six mice in each group. Vaccinations for groups 1–7 were administered intramuscularly twice at a 3-week interval with pGP145_5m_ DNA vaccine co-formulated with PA-MSHA, and Group 8–14 were three-inoculation strategies as delineated in Table 1.

Two weeks following the last immunization, mice were euthanized and spleens were collected for analysis of cell-mediated immune responses by ELISPOT assay. HIV Env-specific antibody titers and antibody avidity were measured by ELISA.

### ELISPOT assay

The ELISPOT assay for HIV-1 Env-specific T-cell responses was carried out according to the protocol provided by the manufacturer with minor modifications (BD ELISPOT Mouse IFN-γ ELISPOT Set and IL-2 ELISPOT Set, BD, San Diego, CA). Briefly, 96-well plates were coated at 4°C overnight with 10 μg/ml of anti-mouse IFN-γ or IL-2 in sterile PBS. The plates were washed four times with 200 μl/well phosphate buffered saline Tween-20 solution (PBST) and blocked with RPMI-1640 containing 10% fetal bovine serum (FBS) at room temperature for 2 h. Splenocytes were seeded into wells with at 5×10^5^ cells/well with 100 μl of envelope (env) peptide (at final concentration of 5 μg/ml) (peptide sequence: C0604200005: CKEVHNVWATHACVPTDPNP, C060420006: SELYKYKVVEIKPLGIAPTA, C0604200007: QQSNLLRAIEAQQHLLQLTV) and incubated in a humidified 5% CO_2_ incubator at 37°C for 24 h. After incubation, ELISPOT plates were developed according to manufacturer's instructions. Finally, plates were air-dried, and the spot-forming cells (SFC) were quantified with a Bioreader-4000 automated ELISPOT reader (BioSys, Karben, Germany) and normalized for 10^6^ splenocytes.

### ELISA assay

96-well flat-bottom plates (Costar, Corning, NY) were coated with purified recombinant gp120 protein (final concentration 0.5 μg/ml) in coating buffer (0.012 M Na_2_CO_3_ and 0.038 M NaHCO_3_, pH 9.6) at 4°C overnight. The gp120 (a recombinant protein of HIV-1 CN54 strain) was expressed in 293T cells and purified to 95% purity. Plates were washed five times with phosphate buffered saline Tween-20 solution (PBST), and blocked with 3% bovine serum albumin (BSA) in PBST at 37°C for 1 h. Sera from each mouse group were sequentially diluted (two-fold) with PBST with starting concentration of 1∶100, and a 100 μl aliquot of the diluted sera was added to each well. After 2 h incubation at 37°C, the plates were washed five times with PBST and then incubated for 1 h with 1∶10000 diluted HRP-labeled goat anti-mouse IgG antibody (Santa Cruz Biotechnology) at 37°C. The plate was washed five times with PBST, and 100 μl of fresh Tetramethylbenzidine (TMB) substrate (Sigma, St. Louis, MO) was added to each well, and incubated for 15 min at room temperature. The reaction was stopped by adding 25 μl of 2 M H_2_SO_4_, when the optical density (OD) was measured at 450 nm with a Multiscan ELISA plate reader (Thermo Life Sciences, Hampshire, United Kingdom).

The IgG isotypes in HIV-1 specific immune response were measured using similar ELISA assay as described above. Binding antibodies were detected by HRP-labeled goat anti-mouse IgG1 and IgG2a antibody (Santa Cruz Biotechnology) at dilution of 1∶5000.

Anti-HIV Env titers were expressed as geometric mean titers ± the standard error of the mean (GMT ± S.E.M.) for each group.

### Avidity ELISA

Avidity ELISA was performed similarly to that of the serum antibody ELISA assay. Samples were diluted to optimized concentrations. Plates were washed three times with 0.05% PBS-Tween 20. Different concentrations of the chaotropic agent and sodium thiocyanate (NaSCN) in PBS were then added (0, 1, 1.5, 2, 2.5, 3 and 3.5 M NaSCN). Plates were incubated at room temperature for 15 min and then washed six times with PBST. Other procedures were similar to ELISA assay as described above. All assays were done in duplicate.

### Statistical analysis

One-way ANOVA analysis was used to compare experimental groups and was followed by non-pairwise multiple comparisons using a Newman-Keuls test. A p-value of <0.05 was considered significant. All statistical calculations were computed with Prism 5.0 software (GraphPad Inc).

In the expression profiling studies, a gene was considered differentially regulated if the difference was ≥3-fold in comparison with the control and markedly differentially regulated if the difference was ≥10-fold.

## Results

### PA-MSHA activated Toll-like receptor pathway in mouse splenocytes

To directly assess the role of PA-MSHA during TLR activation, mouse splenocytes were stimulated with PA-MSHA *in vitro* and differential expression of the TLR pathway molecules were measured at several time-points by real-time qRT-PCR.

**Figure 1:**
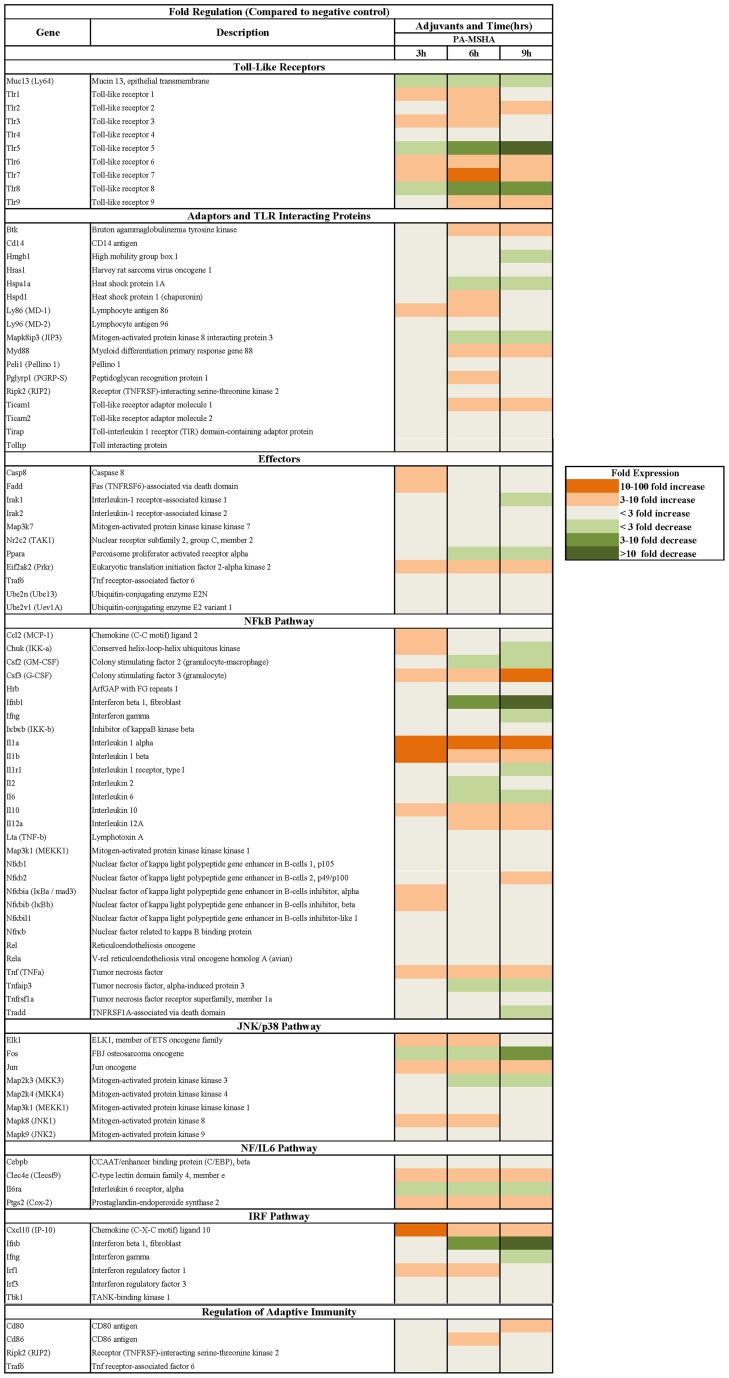
Heat map of the expression of TLR pathway genes after stimulation with PA-MSHA at different time points. Up-regulation was defined as a ≥3-fold increase as compared with negative control, and down-regulation as a ≤3-fold decrease.

Of the 84 genes included in the RT^2^ Profiler PCR Array Mouse Toll-Like Receptor Signaling Pathway kit, 56 (67%) were differentially expressed in the stimulated splenocytes for at least one time point (Fig. 1). The heatmap shows that out of the 84-genes involving TLR signaling pathway, a significant number of molecules were affected by PA-MSHA, including NF-κB/JNK/p38 pathway molecules, effectors and receptor molecules. In aggregate, there was widespread increase in the expression of genes mediating TLR pathway signaling activation at 3 h (expression of 21 genes increased ≥3-fold, and expression of 3 genes increased ≥10-fold), 6 h (expression of 25 genes increased ≥3-fold, and expression of 2 genes increased ≥10-fold) and 9 h (expression of 18 genes increased ≥3-fold, and expression of 2 genes increased ≥10-fold) after stimulation. Decreased expression of genes appeared in a time-dependent manner, with the expression of 5 genes, 11 genes and 16 genes having decreased ≥3-fold at 3 h, 6 h and 9 h respectively. Moreover, 4 and 5 genes were downregulated more than 10-fold at 6 h and 9 h respectively.

The expression of several molecules upstream of these signaling pathways (TLR1, TLR2, TLR3, TLR6, TLR7 and TLR9) increased significantly, and critical adaptors and effectors (MyD88, Ticam1, Nfkb2, and TAK1) were upregulated at various time points. All instances of activation involved the NF-κB, JNK/p38, NF/IL-6 and IRF pathways. Furthermore, among the genes downstream of TLR signaling, the cytokines and proinflammatory factors IL-1, IL-10, IL-12, TNF-α, G-CSF, IP-10 and Cox-2 were increased time-dependently.

**Figure 2:**
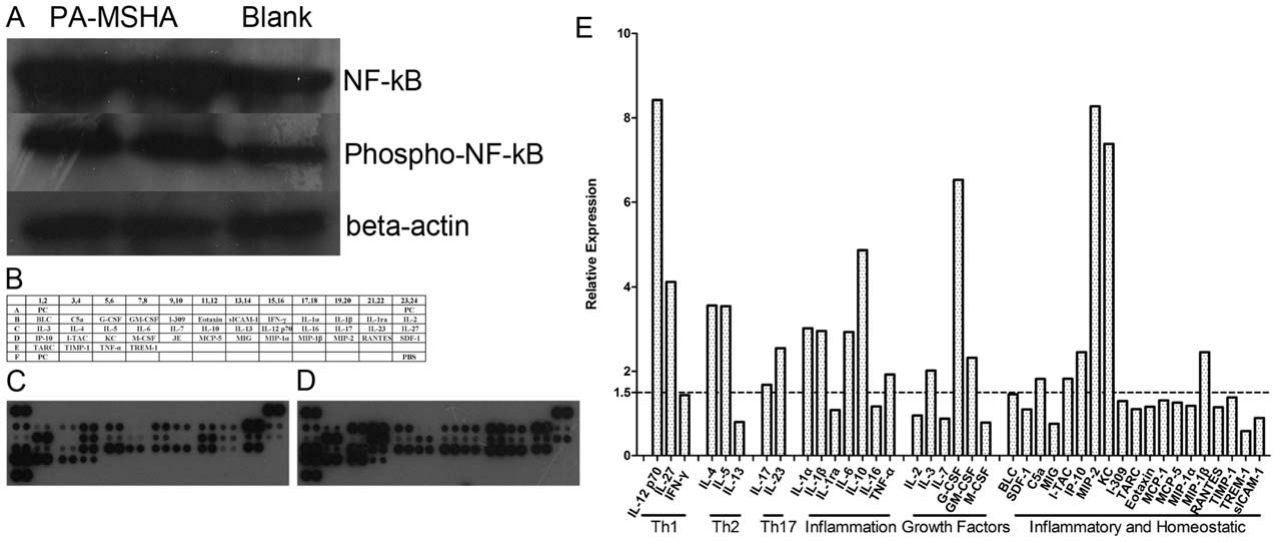
The expression of NF-κB and cytokines involved TLR pathway performed by Western blot and proteome profiling assay. (A) Splenocytes were harvested 6 h after stimulation by PA-MSHA and lysate by RIPA buffer. Expression of NF-κB and phosphor-NF-κB proteins from un-stimulated and PA-MSHA treated splenocytes by Western blot analysis. (B) Spot legend for the cytokine array containing 40 different protein species with duplicates. The array also contains 3 positive control (PC) proteins. For details, see manufacture's protocol (R & D Systems, UK). (C) Negative control for adjuvant stimulation, using medium alone. (D) PA-MSHA (10^7^ CFU/ml) treatment. All arrays were processed under the same conditions after stimulation. (E) Densitometry represents mean corrected for background. Expression levels elevated by more than 1.5-fold relative to control were identified as up-regulated.

Consistent with the result of TLR activation at the RNA level, we confirmed by Western blot assay that the pivotal transcriptional factor NF-κB was up-regulated following stimulation by PA-MSHA (Fig. 2A). Furthermore, several downstream cytokines or chemokines showed significant increase during proteome profiling (Fig. 2B–E), including Th1-type cytokines (IL-12, IL-27), Th2 cytokines (IL-4, IL-5), inflammatory factors (IL-1α, IL-1β, IL-6 and IL-10) and chemokines (IP-10, MIP-2).

The profiling results conclusively demonstrated that PA-MSHA had an effect on the TLR pathway in mice splenocytes, although detailed mechanisms and specific means of interaction between TLR and PA-MSHA remained unclear.

### PA-MSHA up-regulated co-stimulatory molecule CD86 in BMDCs and promoted BMDC maturation

**Figure 3:**
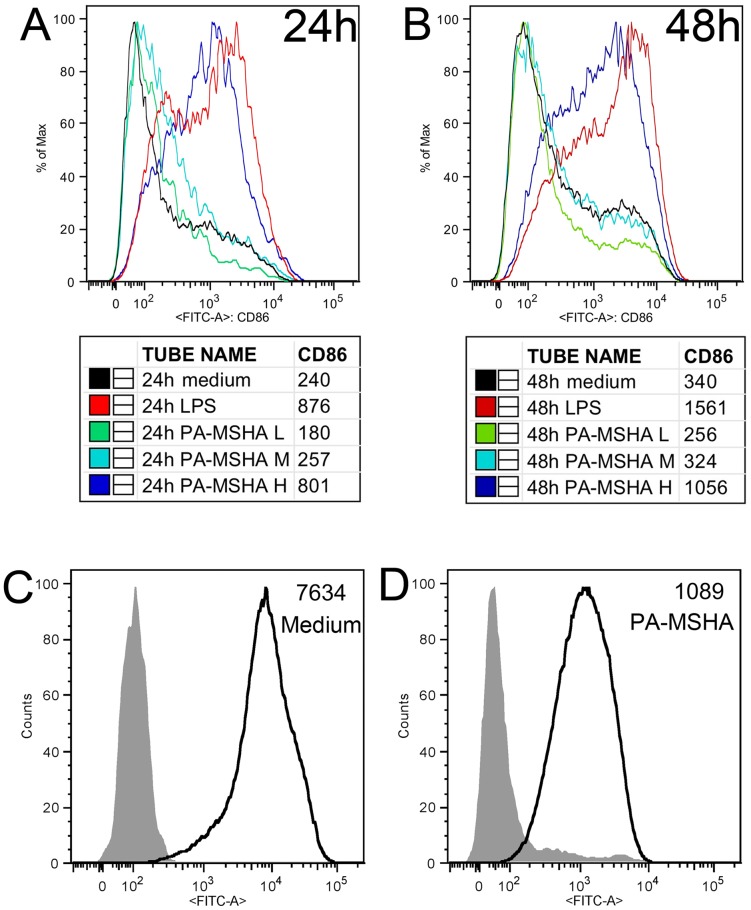
PA-MSHA stimulated up-regulation of co-stimulatory molecules in BMDCs and promoted BMDCs maturation. BMDCs were generated as previously described. Upper panel: Cells were cultured for an additional 24 h (A) and 48 h (B) on day 6 at standard conditions with either PA-MSHA (10^3^,10^5^,10^7^ CFU indicated by L, M and H in the graph, respectively) or LPS (10 ng/ml) and were harvested and analyzed by flow cytometry. Untreated controls are represented by medium alone and positive control by LPS. PA-MSHA induced expression of the surface molecule CD86. The histogram shows CD11c+ -CD86 expression. Lower panel: The endocytosis activity of stimulated BMDCs for 24 h in the absence (C) or presence (D) of PA-MSHA. BMDCs were incubated with 200 μg/ml FITC-Dextran in the dark for 1 h at 4°C (filled histograms) to assess non-specific binding or at 37°C (open histograms) to assess specific uptake. These data presented are from one of three separate experiments.

In this study, we evaluated the role of PA-MSHA in BMDC activation. BMDCs were stimulated with different concentrations of PA-MSHA, control medium or LPS, and the expression levels of co-stimulatory molecules were determined by flow cytometry. BMDCs activated with PA-MSHA (10^7^ CFU) or LPS (10 ng/ml) expressed higher levels of CD86 than BMDCs cultured in the presence of medium only. By contrast, PA-MSHA did not enhance the expression levels of CD86 on DCs at the low (10^3^ CFU) or medium (10^5^ CFU) concentrations at 24 h following stimulation (Fig. 3A) or at 48 h (Fig. 3B). And there was no significant change in the expression levels of MHC-I at various time-points and concentrations (data not shown).

One feature of DC maturation is a reduced ability to uptake due to decreased endocytosis. To determine whether the maturation was accompanied by this hallmark, the endocytic uptake assay was performed. Whereas iDCs are proficient at endocytic uptake, mature DCs reduce the ability of uptake in antigen preparation to T cells [31]. As shown in Fig. 3C–D, we observed a significant reduction in endocytosis in PA-MSHA stimulated BMDC.

The results in the present study suggest that PA-MSHA can cause murine BMDC maturation as assessed by up-regulation of immunostimulatory molecules, and their functional properties appear to be particularly reliant on the dosage strength and number of exposures.

### PA-MSHA enhanced antigen-specific cellular immune response in vivo

**Figure 4:**
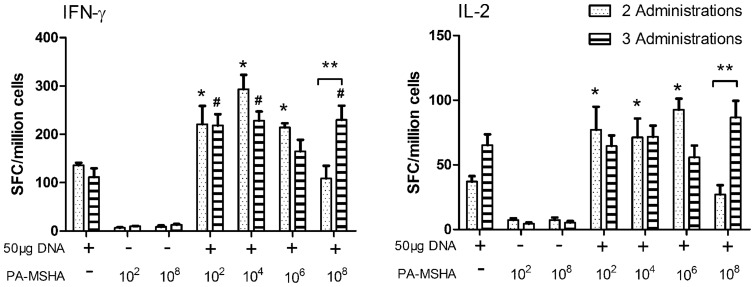
HIV-1 Env specific cellular immune responses as detected by IFN-γ and IL-2 ELISPOT. Mice were immunized for either two or three times at a 3 week interval with an HIV DNA plasmid co-formulated with different concentrations of PA-MSHA adjuvant. * indicates p<0.05 when compared with 50 μg DNA alone in 2 administrations; # indicates p<0.05 when compared with 50 μg DNA alone in 3 administrations; and ** indicates p<0.05 when comparing between 2 and 3 inoculations at 10^8^ CFU dose PA-MSHA.

Cellular response studies indicate that the Env-specific T cell response was enhanced in the two-inoculation regimen at a low PA-MSHA dose (10^2^,10^4^ CFU). Unexpectedly, high doses of PA-MSHA (10^8^ CFU) did not increase specific cellular responses, but in fact even impaired vaccine immunoreactivity in the two-inoculation strategy. After the third vaccination, the high dose group (10^8^ CFU) exhibited the same level of cellular response as the low dose groups (Fig. 4). It is noteworthy that the number of inoculations did not influence the strength of the immune response at low PA-MSHA doses (10^2^, 10^4^ CFU), while three inoculations of high dose PA-MSHA resulted in significantly greater responses compared with two inoculations of 10^8^ CFU PA-MSHA. The exact cause and mechanism for this immunosuppressive effect is currently not understood. The ICS results showed the same tendency as the ELISPOT results (shown in Fig. S1). These results clearly demonstrated that the two-inoculation DNA vaccine strategy resulted in active and robust HIV-specific immunological response when co-administered with low dose PA-MSHA.

### Low dose PA-MSHA enhanced DNA vaccine-induced HIV Env-specific humoral immune response

**Figure 5:**
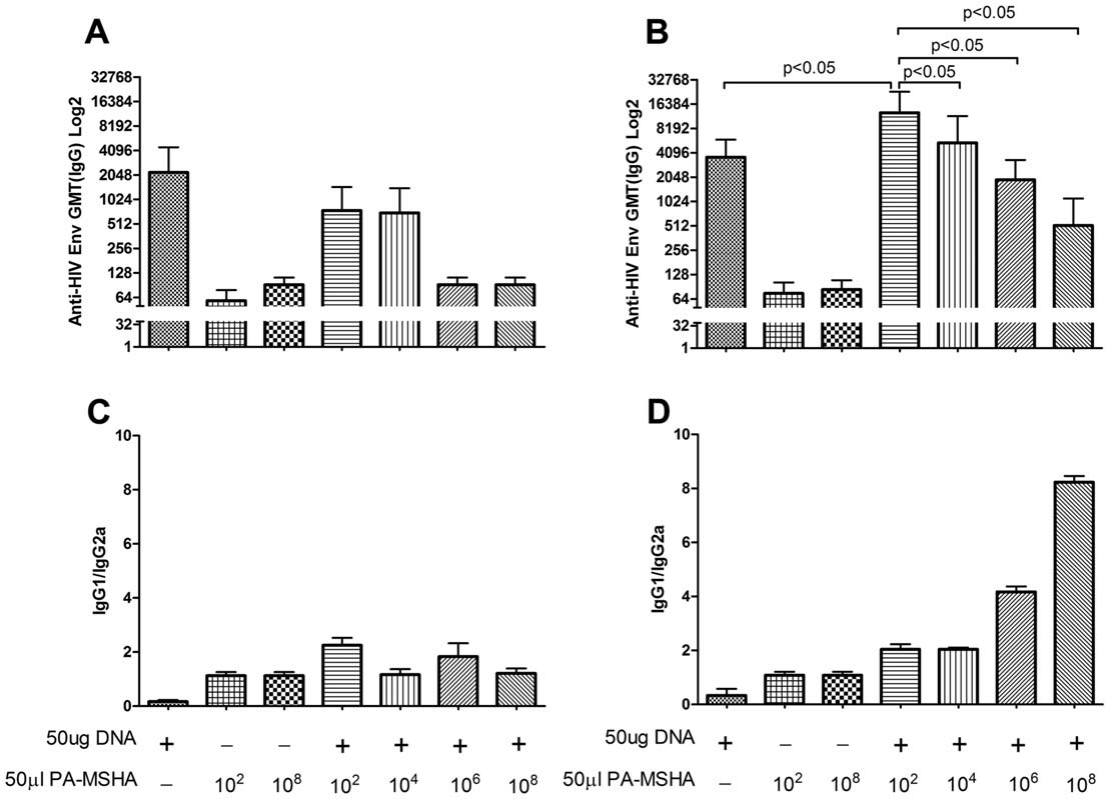
The HIV-1 Env specific humoral immune response as detected by ELISA. (A) and (B) show that the HIV-Env specific antibody titer detected by ELISA after two and three inoculations, respectively. We analyzed IgG isotypes (IgG1/IgG2a) in the humoral response after two (C) and three (D) inoculations.

Humoral immune responses play an essential role in defending against infection. Here, PA-MSHA could not promote the HIV-1 Env-specific antibody response in two inoculations regimen (Fig. 5A), and IgG isotype study showed all groups induced balanced Th1/Th2 responses (Fig. 5C). However, low dose PA-MSHA (10^2^ CFU) significantly increased HIV specific antibody titer (Fig. 5B), in comparison with others PA-MHSA doses in the three-inoculation regimen. High dose PA-MSHA (10^8^ CFU) still showed poor ability to induce humoral responses, but the IgG isotype results indicated 10^8^ CFU PA-MSHA induced Th2-bias immunity responses (Fig. 5D).

### Low dose PA-MSHA promoted antibody avidity of mouse Env antiserum

Antibody avidity was assessed using sodium thiocyanate (NaSCN)-displacement ELISA was used to evaluate the avidity of the antibody [32], [33], [34], [35], in which graded concentrations of NaSCN were used to disrupt the antigen-antibody interaction. The effective concentration of NaSCN required to release 50% of antibodies (ED_50_) from binding is higher for antibodies with stronger avidity for their antigens.

**Figure 6:**
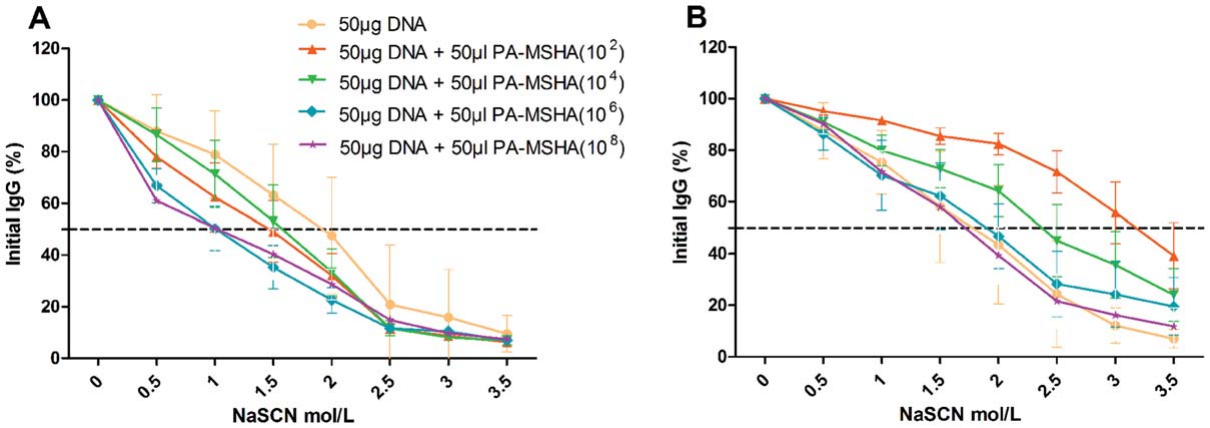
Avidity of the anti-Env IgG raised by the different PA-MSHA concentrations. Sera obtained from inoculated mice were analyzed for 2-inoculation (A) and 3-inoculation regimens (B) in an HIV Env-specific NaSCN-displacement ELISA. Assays used serum samples from each mouse at a dilution of 1∶100. Data points are the average of three independent assays ± standard error.

ED_50_ for antibodies collected from mice two weeks after the most recent vaccination with DNA alone was ∼2.0 M, higher than DNA co-immunization with PA-MSHA in the two-inoculation groups (Fig. 6A). In contrast, in the three-inoculation strategy, the ED_50_ of DNA co-vaccination with 10^2^ CFU PA-MSHA was ∼3.2 M (Fig. 6B), higher than other groups. NaSCN-displacement ELISA demonstrated the advantage of promoting antibody maturation in the three-inoculation strategy with 10^2^ CFU PA-MSHA (Fig. 6B). However, low dose PA-MSHA is capable of inducing both robust humoral immune response and high avidity antibody in the three-inoculation strategy and may serve as the best adjuvant strategy.

## Discussion

In this study, we assessed the ability of PA-MSHA to activate innate immune responses in murine splenocytes and BMDCs, as well as its *in vivo* adjuvant effects in enhancing cellular and humoral immune responses to HIV-1 Env peptides following co-administration with a DNA vaccine. PA-MSHA enabled activation of the TLR pathway mediated by NF-κB and JNK signaling in splenocytes, and promoted the up-regulation of co-stimulatory molecule CD86 in BMDCs. As well, co-inoculation of the DNA vaccine with low dosages (10^2^–10^4^ CFU) of PA-MSHA enhanced specific immunoreactivity against HIV-1 Env in both cellular and humoral responses, and promoted antibody avidity maturation. However, high doses of adjuvant (10^8^ CFU) resulted in an immunosuppressive effect; a two- or three-inoculation regimen yielded low antibody responses and the two-inoculation regimen exhibited only a slight cellular immunity response. To our knowledge, this is the first report demonstrating the utility of PA-MSHA as an adjuvant to a DNA vaccine.

In the *in vitro* assay, we hypothesized that PA-MSHA would have a stimulatory effect on murine cells similar to the effects of bacterial flagellin. Flagellin has been shown to trigger TLR pathway activation through its interaction with TLR5 [22], [23]. Here, we found that PA-MSHA resulted in a decrease in TLR5 level, but significant upregulation of TLR1, TLR2, TLR3, TLR6, TLR7, TLR9, MyD88, and associated adaptor molecules (Fig. 1). This suggests that PA-MSHA can successfully trigger TLR pathway activation and upregulation of cytokines and proinflammatory factors independent of TLR5. In addition, PA-MSHA also resulted in substantial upregulation of CD86 in BMDCs and promoted BMDC maturation. As the whole bacterial PA-MSHA constitutes many potential immunogenic components, further studies are needed to pinpoint the exact components and mechanisms that facilitate the adjuvant effects in splenocytes and each different cell type.

In the *in vivo* study, we tested four concentrations of PA-MSHA and two vaccination schedules. Strong cellular immune response as measured by IFN-γ and IL-2 ELISPOT were elicited from low doses of PA-MSHA for both the two- and three-inoculation strategies, with no significant difference between the two. The strong response of the two-inoculation schedule at low dose is particularly notable, as this was a more robust performance than found in other adjuvants similarly tested in our laboratory previously. However, high dosages of PA-MSHA were ineffective and sometimes even detrimental for promoting immune responses. Within the three-inoculation strategy, high dose PA-MSHA (10^8^ CFU) promoted specific cellular responses up to the same strength as low-dose groups, while humoral responses were augmented only for the lowest-dose group (Fig. 4 and Fig. 5B). Similarly, within the two-inoculation regimen, cellular responses were attenuated for the high dose group, while humoral responses were not enhanced for any adjuvant dose (Fig. 5A).

The dose dependency of both cellular and humoral responses may be due to certain immunosuppressive or tolerance-eliciting properties of PA-MSHA. Previous studies have shown that PA-MSHA to inhibits cellular proliferation and induces apoptosis in human breast cancer cell lines in a dose-dependent manner [24] through EGFR pathway signaling [36]. High concentrations of PA-MSHA (3.6–6×10^8^ CFU) induced high levels of apoptosis and proliferation inhibition [24]. Therefore, it is possible that high dose PA-MSHA also inhibited normal immunological cell proliferation in our assays here through EGFR signaling. And our other data also demonstrated the immunosuppressive in other different immunization strategies (shown in Fig. S2). Meanwhile, the enhancement of cellular immune responses (compared to DNA vaccine alone) in the high dose, three-inoculation group may be due to a yet unknown mechanism.

In addition, IgG isotype assay also suggests that high dose PA-MSHA induces immune tolerance or immunosuppression responses. Our results showed that a dose range of PA-MSHA from 10^2^ to 10^6^ CFU induced Th1/Th2 type immune responses in balance (Fig. 5C/5D). However, a typical Th2 type response was induced by 10^8^ CFU of PA-MSHA in the three inoculations regimen, which would be expected to elicit an antibody biased response; yet in reality, the 10^8^ dose could not enhance a humoral response. This therefore raises the question of why the Th2-type immune response resulted in a low humoral response. As inactivated bacterium, PA-MSHA is a powerful pathogen and is involved in the activation of naïve immunity, which may also contribute to negative immunological regulation at high dose during early stages of immunization. However, further studies are needed to clearly characterize Th2-type activity by ELISPOT.

Finally, the vaccination schedule also had an effect on the adjuvant properties of PA-MSHA. The addition of the third inoculation most clearly improved humoral responses, particularly in low-dose groups, rather than cellular responses. While all dose levels failed to enhance humoral responses in the two-inoculation strategy, three inoculations of 10^2^ CFU PA-MSHA induced the strongest antibody response, and even 10^8^ CFU PA-MSHA retained a low humoral immune response. As well, antibody maturation was clearly enhanced by the third inoculation, as seen in the increased antibody avidity (Fig. 6 B).

In sum, the immune response profile and antibody avidity of low doses of PA-MSHA (10^2^ or 10^4^ CFU) makes it an attractive candidate strategy for use as an HIV-1 DNA vaccine adjuvant. In particular, the difference in immune response at two inoculations (a predominantly cellular response) and at three inoculations (a predominantly humoral response) suggests potential in tailoring the adjuvant strategy to the particular needs of a given vaccine, disease, or patient. It may also be useful for employing PA-MSHA in a combination of multiple adjuvants to control different immune responses at various time-points in the vaccination schedule. A vaccine regimen pre-designed to incorporate several adjuvants according to their particular characteristics may provide a promising direction for future HIV vaccine research.

## Supporting Information

Figure S1 **Env-specific cellular immune responses as detected by intercellular cytokines assay.** Mice splenocytes were isolated as described previously, and washed twice with phosphate-buffered saline (PBS) containing 2% bovine serum albumin (BSA). Then, cells were seeded to wells at 1×10^6^/ml and were incubated for 6 h at 37°C and 5% CO2, with 100 μl Env peptide pool containing 5 μg/ml of each individual peptide, as described previously. After that, cells were stained with anti-mouse CD3-PerCP and anti-CD8a-FITC antibodies. After permeabilization, cells were stained for intracellular anti-IFN-γ-APC and anti-IL2-PE and fixed. After washing twice, samples were resuspended with PBS and immediately analyzed on a FACS Aria cytometer.
(TIF)

Figure S2 **Env-specific IFN-γ T-cells and antibodies induced by co-administration of DNA vaccine and adjuvant.** Data are shown as mean ± SD (n = 6 mice/group). After the third immunization, IFN-γ production of splenic lymphocyte was determined by ELISPOT assay (Fig. S2A) and Env-specific binding antibody was determined by ELISA (Fig. S2B). Six- to eight-week-old female BALB/c mice (Vital River Laboratories) were randomly divided into 5 groups with six mice in each group. Group 1∶50 μg pDVRI1.0-gp145_5m_ +50 μl PBS; Group 2∶50 μl 10^8^ CFU PA-MSHA +50 μl PBS; Group 3∶50 μg pDVRI1.0-gp145_5m_ +50 μl 10^8^ CFU PA-MSHA in separated legs; Group 4∶50 μg pDVRI1.0-gp145_5m_ +50 μl 10^8^ CFU PA-MSHA in premixed; Group 5∶50 μg pDVRI1.0-gp145_5m_ +50 μl 10^8^ CFU PA-MSHA simultaneously.
(TIF)
